# Does Drug Holiday Affect Jaw Trabeculation in Medication Related Osteonecrosis of the Jaw? – A Pilot Study

**DOI:** 10.4317/jced.59503

**Published:** 2022-04-01

**Authors:** Niranzena Panneer-Selvam, Abrar Alamoudi, Joseph Riley III, Joseph Katz

**Affiliations:** 1 MDS. Resident, University of Florida College of Dentistry, Gainesville, FL, USA. Assistant Professor, Creighton University School of Dentistry, Gainesville, FL, USA; 2 BDS. Resident, University of Florida College of Dentistry, Gainesville, FL, USA; 3 PhD. Professor, University of Florida College of Dentistry, Gainesville, FL, USA; 4 DMD. Professor, University of Florida College of Dentistry, Gainesville, FL, USA

## Abstract

**Background:**

The role of drug holiday in long term antiresorptive drug users to prevent MRONJ is debated for quite some time. We aimed to compare jawbone trabeculation between patients on drug holiday with those not on drug holiday among Medication Related Osteonecrosis of the Jaw (MRONJ) cases using fractal analysis and to estimate the frequency of MRONJ despite being on drug holiday.

**Material and Methods:**

The sample of 18 MRONJ cases were divided into drug holiday, and non-drug holiday groups. Non-drug holiday group was further divided into pre- and post- drug holiday groups. Jawbone trabeculation was assessed utilizing fractal analysis method (ImageJ software) by two observers. ANOVA was used to compare the fractal dimension (FD) values between the groups. A ‘*p*’ value of less than 0.05 was considered significant.

**Results:**

8 patients developed MRONJ despite being on drug holiday (44.44%). There was no significant difference in the FD values between drug holiday and pre-drug holiday groups. When pre- and post-drug holiday FD values were compared, the difference was significant only on the right maxilla with observer 2. Also, no significant difference was noted between the two observers.

**Conclusions:**

Bone trabeculation remained unaltered following drug holiday from anti-resorptive drugs. This study serves as a preliminary proof for the argument against drug holiday which portrays the necessity for a detailed prospective study in this arena.

** Key words:**Osteonecrosis, bisphosphonates, Bisphosphonate-Associated Osteonecrosis of the Jaw, anti-resorptive drugs, osteoporosis, jaw.

## Introduction

Medication-related osteonecrosis of the jaw (MRONJ) has been established as a serious complication associated with anti-resorptive drugs, mainly bisphosphonates (BP) and receptor activator of NF-kB ligand (RANKL) inhibitors, antiangiogenic agents, and mammalian target of rapamycin (m-TOR) inhibitors (1). The list of drugs that could cause MRONJ as a side effect is ever growing. Intravenous BP are widely used to cope with cancer-related conditions like hypercalcemia related to malignancy, skeletal related events (SRE) associated with bone metastasis and lytic lesions of multiple myeloma (2). Oral BP and certain IV BP such as once yearly infusion of zolendronate and quarterly infusion of ibandronate, have been approved for management of osteoporosis (2). Antiangiogenic drugs have found their use in the treatment of renal cell carcinomas, gastrointestinal tumors, and neuroendocrine tumors (2). Increasing use of these drugs in the medical field has caused a surge in the complications associated with them. The cumulative incidence of MRONJ among cancer patients under zolendronate (BP drug) has be estimated to be in the range of 0.7 to 6.7% (2). The incidence of MRONJ among patients on denosumab (RANKL inhibitor) is found to be around 1.7% and the risk ranges from 0.7 to 1.9% (2,3).

Several risk factors have been identified for the development of MRONJ. Medication related risk factors including the underlying medical condition, and type and duration of the medication received, local factors including tooth extraction, and anatomic location, periodontal disease, and demographic, systemic and genetic factors interplay to develop this complication (2). Among these, dentoalveolar surgery, especially tooth extraction is a common predisposing factor. The risk of developing MRONJ following tooth extraction among cancer patients who are on IV BPs ranges from 1.6 to 14.8% (2,4,5). However, pre-existing inflammatory conditions like periapical pathology or periodontal disease may also contribute to it (2). To prevent the occurrence of MRONJ, the concept of drug holiday before invasive dental procedures was advocated. Around half the amount of serum BP would undergo renal excretion. Osteoclasts with a life span of two weeks would serve as the next major reservoir. Thus, free serum BP would be extremely low two months post the last dose, making two-month period of drug holiday before any invasive dental procedure adequate to prevent the adverse effects (6). This is in accordance with the AAOMS committee who recommended a modified drug holiday strategy in patients with the history of oral BP usage over 4 years (2). However, this concept is still controversial. The skeletal binding sites for BP are unsaturable. This, in turn, leads to huge accumulation of BP in the bone. Therefore, even after stopping the drug for few months, the bone could act as a reservoir releasing BP continuously for a period of around 10 years (7,8). Even the advisory committee from the ADA Council on Scientific Affairs and the international consensus paper did not recommend any drug holiday before invasive dental procedures (9,10). However, the short half-life of denosumab is quite low, making drug holiday more meaningful (8). This controversy on drug holiday formed the basis for our study. Investigating the changes that occur in the bone architecture of the jaws following drug holiday could enlighten us more on this concept.

To our knowledge, till date no studies have analyzed the effect of drug holiday in the trabecular pattern of the jaws in MRONJ patients. We hypothesized that drug holiday in patients on long term anti-resorptive drugs changes the trabecular pattern of the jaws making it less sclerotic thereby reducing the occurrence of MRONJ. The aim of the study was to compare the trabecular pattern of the jaws between patients on drug holiday with those who were not on drug holiday among MRONJ cases and to estimate the frequency of MRONJ despite being on drug holiday.

## Material and Methods

This retrospective study was approved by our university review board under exempt and is in compliance with the Helsinki Declaration. Also, we have complied with the STROBE checklist for non-randomized studies. A total of 24 MRONJ cases were identified from January 2011 to April 2019. The list of patients was provided by the University’s Integrated Data Repository (IDR). MRONJ patients diagnosed according to the AAOMS criteria who were/are on intravenous BP or denosumab were included under the study. Patients on Parathyroid hormone replacement therapy, and those without relevant radiographs were excluded. Following the above criteria, 18 cases qualified for the study.

**Figure 1:**
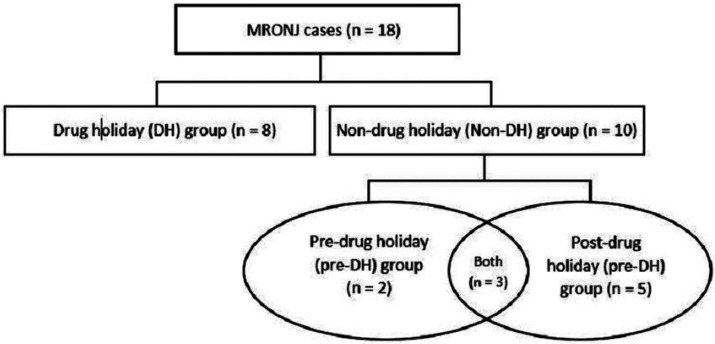
Group design of study samples.

We divided the sample into two major groups: Drug holiday (DH) group (n = 8) consisting of patients who are/were on drug holiday at some point of time before the development of MRONJ; and Non-drug holiday (Non-DH) group (n = 10) consisting of patients who are/were not on drug holiday before the development of MRONJ. But the patients under non-DH group were eventually put on drug holiday after the development of MRONJ. Accordingly, this group (Non-DH) was divided further into two groups: Pre-drug holiday (pre-DH) before stopping the drug (n = 5), and Post-drug holiday (post-DH) after stopping the drug (n = 8). Thus, pre-DH group is considered the true Non-DH group since during this period, the patient was not on drug holiday. Also, pre-, and post-DH groups were not mutually exclusive as some of them satisfied the criteria for both the subgroups since they had relevant radiographs when they were both on and off the offending drug. The layout for the group design is represented in Figure 1. The following information were recorded for all the patients: type of cancer, duration of anti-resorptive therapy, duration of drug holiday, length of time elapsed from the start of drug holiday and the subsequent pantomograph (wherever applicable), and location of MRONJ.

**Figure 2:**
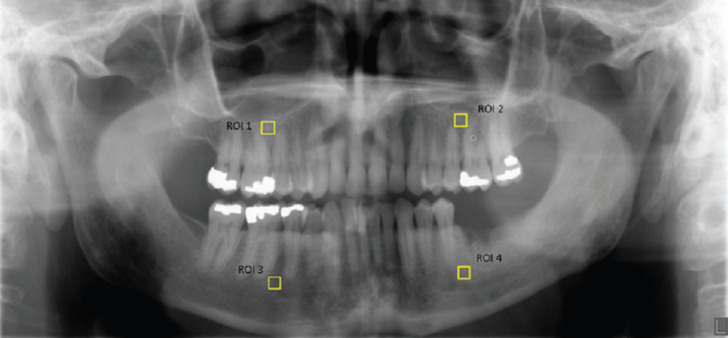
Pantomograph showing the selection of region of interests (ROI).

**Figure 3:**
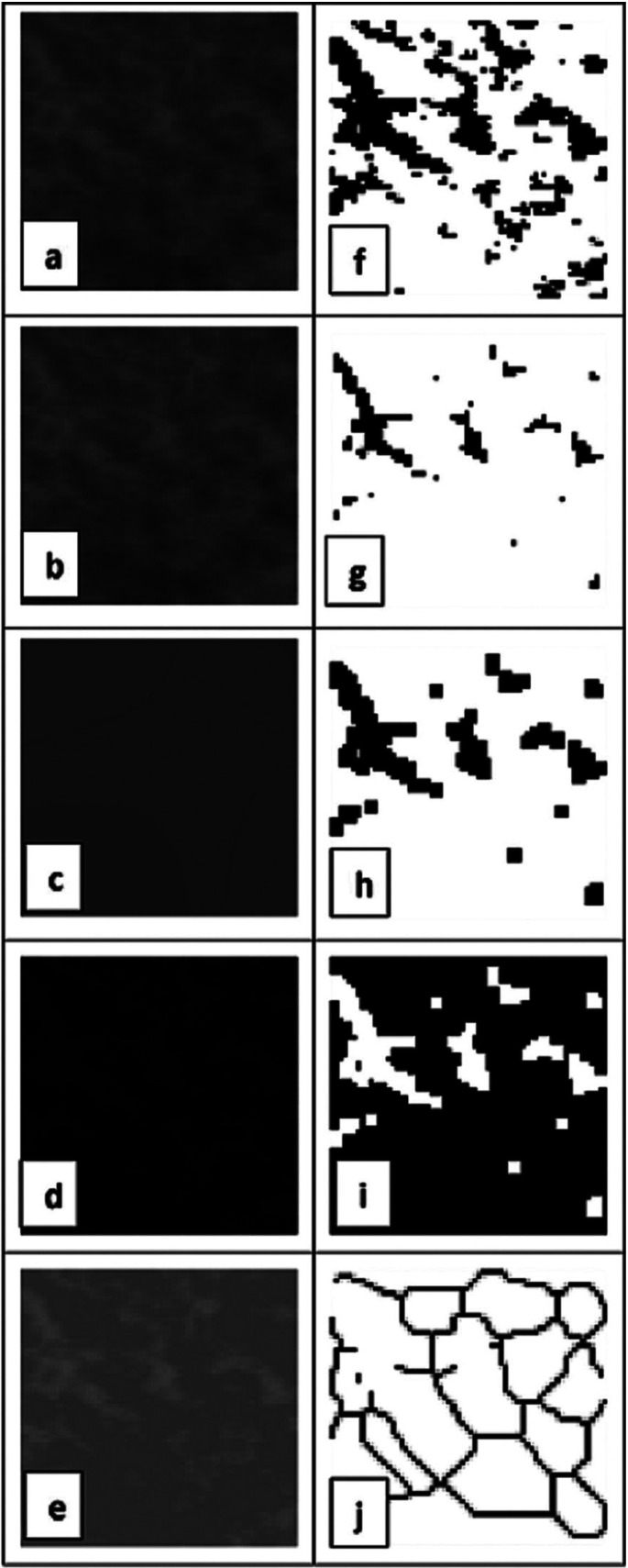
Steps in the image preparation for the measurement of fractal dimension, a. Region of interest (ROI), 64 × 64-pixel image; b. Duplicated image of ROI; c. Blurred image using σ = 35; d. Subtracted image, blurred image subtracted from original image; e. Resultant image with added gray value of 128; f. binarized image; g. eroded image; h. dilated image; I. inverted image; skeletonized image from which FD value was calculated.

**Figure 4:**
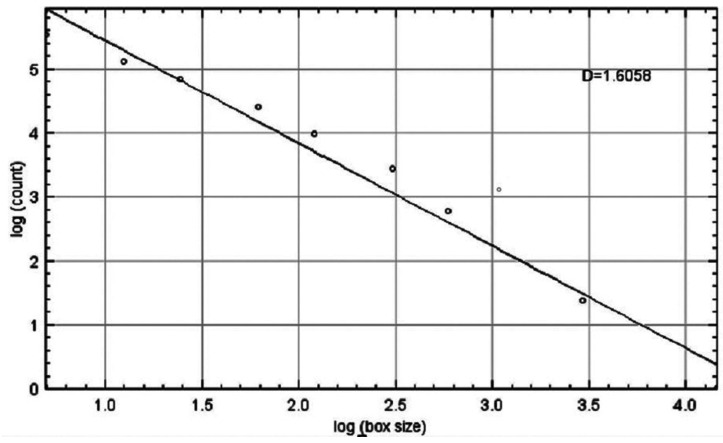
Graph of fractal analysis from which final ‘D’ value (FD) was calculated using box counting method.

The pantomographs (Kodak Carestream CS-8100 2D, Carestream Dental, Atlanta, GA, USA) of the patients belonging to the above groups were analyzed for the bone quality utilizing fractal analysis method using ImageJ software (ImageJ version 1.52p, NIH, Bethesda, MD, USA). The exposure parameters for the pantomographs were set at 70 kV, 10 mA, and 19 s. Under DH and post-DH groups, only pantomographs acquired at least 3 months after the start of drug holiday was analyzed to allow the bone changes to be evident radiographically. The relevant radiographs were anonymized and stored in DICOM format. The measurements were performed by two oral and maxillofacial radiologists (NP, and AA with 7 and 5 years of experience respectively). The selection of regions of interest (ROI) and steps involved in the fractal analysis were discussed between the two observers and a consensus was achieved. The measurements were done based on the methods described by Gaalaas *et al*. (11).

a. Site selection: Measurements were done in the second premolar-first molar region in the maxilla and mandible bilaterally. If the patient was edentulous, the above-mentioned site was selected approximately based on the anatomic landmarks. Only the trabecular bone was included, excluding the cortical bone, lamina dura, MRONJ affected sites and superimposition of the airway or ghost images. Region of interests (ROI), 64 × 64-pixel images, were selected as apically to the adjacent teeth as possible and superior to the inferior alveolar canal or inferior to the maxillary sinus (Fig. 2).

b. Image processing: As the first step, using the histogram stretching tool, the pantomographs were standardized by including 0 to 255 gray scale. Then, with the help of crop tool in ImageJ, 64 × 64-pixel images were cropped from all the four ROIs in the original image (4 images in total). The cropped image was duplicated and blurred using Gaussian blur function (σ = 35). The blurred image was then subtracted from the original image. A value of 128 was added to the resultant image. This image was then binarized, eroded and dilated once. Images were then inverted and skeletonized (Fig. 3).

c. Calculation of fractal dimensions: Fractal dimensions were measured using the ‘ImageJ Fractal box count’ function. The box sizes were set to 2, 3, 4, 6, 8, 12, 16, 32 and 64 pixels. The ‘D’ value was noted (Fig. 4). The mean ‘D’ value was calculated separately for the above-mentioned specific sites. This value will be referred to as fractal dimension (FD) value.

-Statistical analysis.

ANOVA was used to compare the mean ‘D’ values between DH and pre-DH groups, and pre-DH and post-DH groups respectively. Paired ‘t’ test was used to compare the ‘D’ values between the two observers. Pearson’s correlation was used to analyze the correlation between duration of drug holiday (DH time), and time interval between start of drug holiday and radiograph (DH_R) with fractal dimensions respectively. The analysis was done using SPSS version 26. ‘*p*’ values less than 0.05 were considered statistically significant.

## Results

Among the study population (n = 18), 15 patients were on intravenous bisphosphonates and 3 patients on Denosumab. The disorders for which the medication was prescribed were as follows: multiple myeloma (n = 8), prostate cancer (n = 1), metastasis (n = 3), osteoporosis (n = 2), breast cancer (n = 2), squamous cell carcinoma of tongue (n = 1), and breast cancer with osteoporosis (n = 1). The duration of antiresorptive therapy ranges from 3 months to 96 months (Mean ± SD = 33.66 ± 26.11). The MRONJ affected sites were as follows: Right maxilla (n = 3, 16.67%), left maxilla (n = 1, 5.5%), right mandibular region (n = 7, 38.89%), and left mandibular region (n = 8, 44.4%). The time interval between start of drug holiday and the pantomograph ranges from 3 to 24 months (mean ± SD = 10.6 ± 7.4).

Out of 24 MRONJ patients (initially before exclusion), nine developed MRONJ despite being on drug holiday (37.5%). When the FD values between the DH group and pre-DH groups were compared, there was no statistically significant difference (*p* > 0.05) (Table 1). On comparison of FD values between pre-DH and post-DH groups, there was only significant difference among FD21 (FD on the right maxilla with observer 2) (*p* < 0.05) (Table 2). To calculate the interobserver reliability, Pearson’s correlation was used which did not show significant correlation between the FD values of the two observers (*p* > 0.05). But this could be due small sample size. Subsequently, paired ‘t’ test was done where no significant difference was found (*p* > 0.05) (Table 3). When duration of drug holiday and FD values where correlated, there was significant positive correlation with respect to only FD11 (FD on the right maxilla with observer 1) and FD22 (FD on the left maxilla with observer 2) (*p* < 0.05) (Table 4). In all other scenarios, the correlation was not significant. There was no significant correlation amid the time interval between the start of drug holiday and acquiring radiograph (pantomograph in our case) and FD values among group 1 (*p* > 0.05) (Table 5).

**Table 1:**
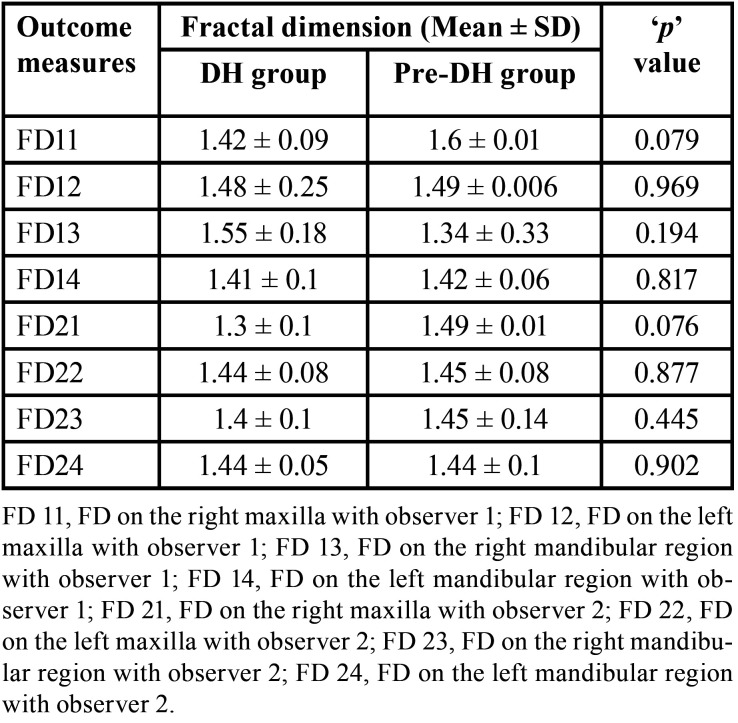
Comparison of fractal dimension (FD values) between Drug holiday (DH) group and Pre-drug holiday (pre-DH) group.

**Table 2:**
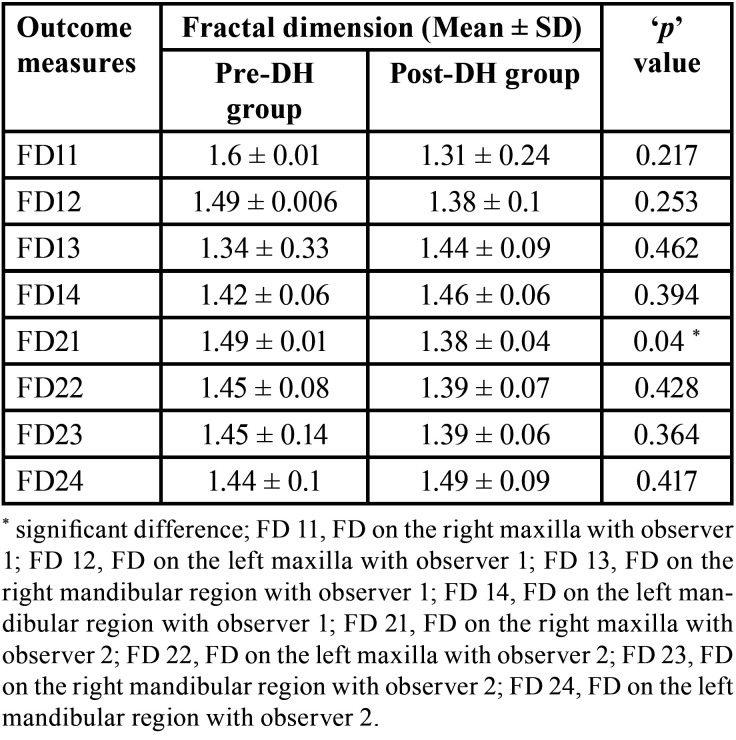
Comparison of fractal dimension (FD values) between Pre-drug holiday (pre-DH) group and Post-drug holiday (Post-DH) group.

**Table 3:**
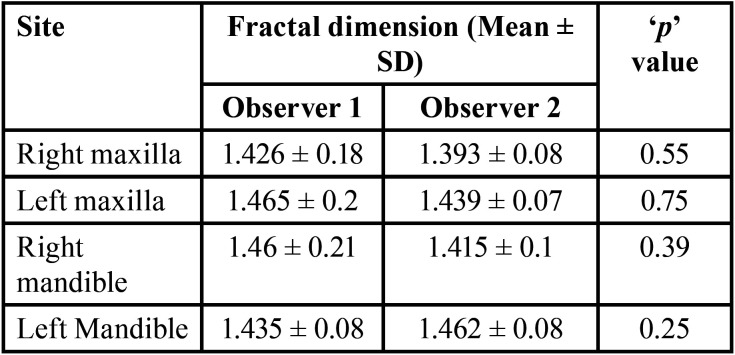
Comparison of Fractal dimensions between Observers 1 and 2.

**Table 4:**
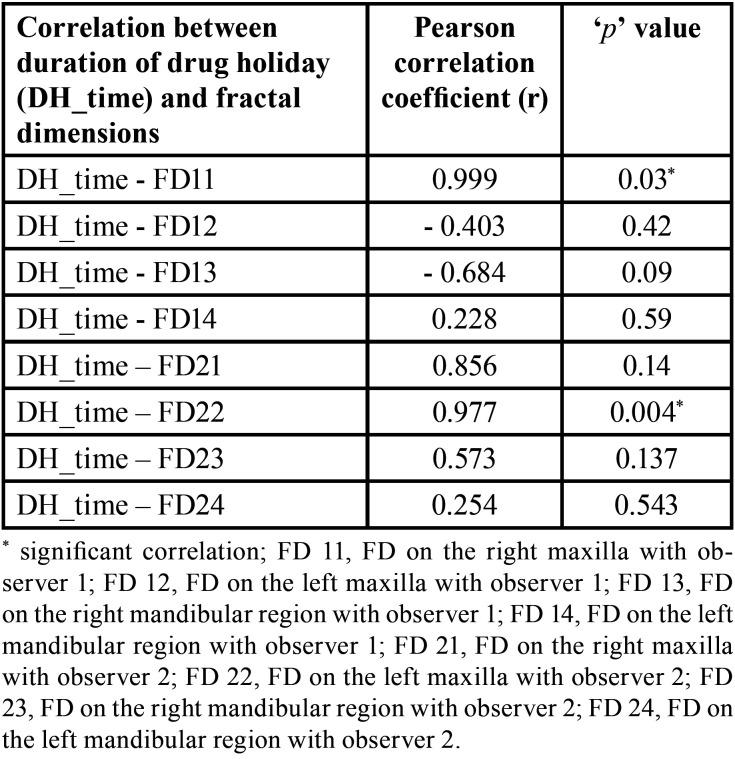
Correlation between duration of drug holiday and fractal dimensions among group 1.

**Table 5:**
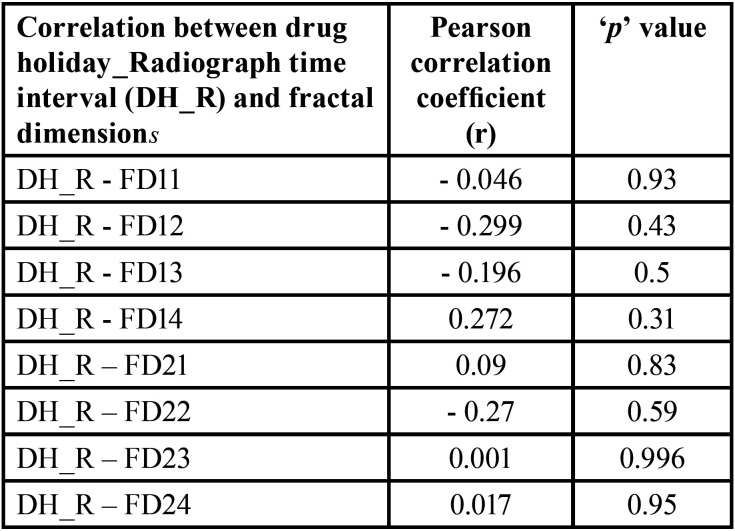
Correlation amid time interval between start of drug holiday and radiograph, and fractal dimensions among group 1.

## Discussion

The beneficial effects of anti-resorptive drugs rely mainly on their capacity to reduce bone turnover rate, making the bone more fracture resistant. Bisphosphonates reduce bone resorption by inhibiting osteoclastic function and inducing their apoptosis by inhibiting farnesyl pyrophosphate synthase, an enzyme in the HMG-CoA reductase pathway (7). They have high affinity for bone matrix. Once incorporated into the bone matrix, the drug effect is seen for more than 10 years (12). Thus, a substantial amount could be accumulated in the bone, which could be constantly released over a period of months or years post drug holiday (13). As the release is partly dependent on bone turnover, the amount released in the presence of bisphosphonate might be fairly low (7). Thereby, it is understood that when the drug is ceased with the aim of reducing its side effects especially MRONJ, there might be continued presence and release of bisphosphonate from the bone leading to lingering antifracture effect during drug holiday. This holds value for patients on low/mild risk of fracture (7). But, for patients who are at high risk of fracture may not benefit from this lingering effect and drug holiday pertaining to this group becomes questionable. Contrarily, the residual drug effect might itself lead to MRONJ, making no difference between drug holiday and those on continued therapy. Analyzing the bone architecture in patients who are on drug holiday would provide more insight regarding the usefulness of drug holiday, whether there is any change after stopping the drug.

On the other hand, the mechanism of action of RANKL inhibitors is quite different, even though the effect on bone turnover is the same. RANKL inhibitors such as denosumab prevent the binding of receptor activator of nuclear factor-κβ ligand (RANKL) in the cell membrane of osteoblasts to the receptor activator of nuclear factor-κβ (RANK) receptors on the osteoclast cell membrane and its precursors, thus inhibiting the development, activation and survival of osteoclasts (14,15). In contrast to bisphosphonates, the serum half-life of RANKL inhibitors are much shorter, around 25-29 days. (15) Another major difference is that they are not incorporated into the bone matrix (16). Thus, drug holiday holds more sense when compared with bisphosphonates as the lingering effect due to substantial release of the drug from bone reservoir is absent.

Eight out of 18 patients in our study developed MRONJ even after stopping the offending drug (bisphosphonates and/or RANKL inhibitors). Out of these, two patients were on denosumab (RANKL inhibitor). This evidence questions the usefulness of drug holiday, especially in patients with high risk of fracture where the benefits of continuing antiresorptive drugs might overweigh the risk. Even though drug holiday appears to be more beneficial with respect to RANKL inhibitors, our results warrant further in-depth analysis of the effect of drug holiday in long term RANKL inhibitor users.

In our study, we used the method of fractal analysis with box counting to analyze the bone architecture in panoramic radiographs. Cancellous alveolar bone is composed of complex interconnection of bony trabeculae which vary in thickness and orientation. Fractal analysis is a non-invasive technique that has shown promising results for analysis of such complex bone architectures (17). Significant correlation has been reported between microcomputed tomography values, the gold standard for measuring trabecular bone, and CBCT FD values (18,19). Also, it has been suggested that FD can be measured reliably on panoramic radiographs. (20) However, exposure time, resolution, and compression of the images might affect the FD values in 2D imaging (17). In our study, since the above factors were standardized, these limitations were eliminated.

There was no significant difference when the FD values were compared between the DH group and pre-DH (Non-drug holiday) group. This implies that there is no substantial alteration in the trabecular bone pattern between patient who were on drug holiday, and those who were not on drug holiday. This is further supported by the absence of significant difference when pre-DH and post-DH FD values were compared within the same subjects at different time period. The only exception was right maxilla FD values with observer 2. But this difference might be owing to the difficulty in selecting the ROI in the maxilla due to the superimposition of the maxillary sinus in few radiographs. Based on the above findings, our initial hypothesis that ‘drug holiday makes the trabecular pattern less sclerotic, thereby reducing the occurrence of MRONJ’ was rejected. Diab *et al*. was favoring continuing the drug without drug holiday for high risk fracture subjects depending on individual patient circumstances and assessment of risk and benefit (7). In the extension of the alendronate Fracture Intervention Trial (FLEX), Black *et al*. found out that bone turnover markers increased after stopping the drug and observed significantly low vertebral fractures in patients who continued the medication (21). In the clinical trial on zolendronate by Black *et al*. in 2012, only small differences were observed in bone density and bone turnover markers in patients who continued for 6 years versus those who stopped therapy after 3 years. In addition, there was significantly fewer vertebral fractures in the group that continued treatment versus those who discontinued therapy. They suggested that patients with high-risk fractures may benefit from continued treatment (22) This was supporting our results. However, when the same clinical trial was extended for another 3 years, there was a non-significant increase in the bone turnover markers in those who discontinued after 6 years compared with those who continued for 9 years. The number of fractures was low and did not significantly differ by treatment. They suggested that almost all patients who have received six annual zolendronate infusions can stop medication for up to 3 years with apparent maintenance of benefits (23). This was contradicting with our study. Similarly, in the extension of the risedronate VERT-NA study, the bone mineral density decreased, and bone turnover markers increased during drug holiday with reduction in the vertebral fractures by 46% when compared with the placebo group (24).

A comparison between the FD values of patients pre- and post-drug holiday with respect to RANKL inhibitors alone, or between bisphosphonate and RANKL inhibitor users could not be made in our study due to low sample size of patients taking denosumab (n = 3) and the lack of appropriate radiographs fulfilling our selection criteria in this group.

According to the present study, there was no correlation between the FD values, and the time interval between the start of the drug holiday to the time when the pantomograph was acquired. In addition, except for certain sites in the maxilla, no significant correlation was noted between duration of drug holiday and FD. Ideally, if our hypothesis of drug holiday was true, over time after stopping the drug, the bone would have become less sclerotic. But this was not the case here as the FD values were unaffected. One reason could be due the reservoir theory of antiresorptive drugs where the bone with unsaturable drug binding sites continuously releases the drug into the blood, thus maintaining its level similar to pre-drug holiday. This in turn maintains the bone architecture in the sclerotic state without allowing any change. However, this absence of correlation could have also been due to relatively small sample size. The positive correlation that was observed in certain maxillary sites could be linked to the less dense trabecular pattern in the maxilla when compared with the mandible.

Being retrospective, there were certain limitations in our study. First, since the study had stringent protocols to follow, there were very few subjects that fulfilled our criteria. Subsequently, our sample size was low under each group. Second was the use of panoramic radiographs. Even though the structural changes of the trabecular bone can be better assessed with CBCT, this is not a routine imaging method. Third, the type of disorder that led to the use of medication, the use of two different medications - intravenous bisphosphonates and denosumab, and comorbid conditions like diabetes, anemia, and chronic corticosteroid therapy could act as confounding factors. Despite these drawbacks, the very fact that 44.44% patients developed MRONJ during drug holiday proves to be solid evidence for the argument against drug holiday. This preliminary finding paves its path to more elaborate prospective studies to find out whether the findings of the study are generalizable with respect to the use of anti-resorptive agents in clinical practice.

## Conclusions

In conclusion, there was no alteration in the trabecular pattern of the jaws following drug holiday from anti-resorptive drugs. This could be attributed to the lingering drug reservoir effect. Our study serves as an initial proof for the fact that benefit of continuing the medication might outweigh the risk of drug holiday to avoid the complications, especially when high risk fracture patients are considered. Even though our study serves as a proof of concept for negating drug holiday, it strongly substantiates the necessity for further prospective studies to discover the role of drug holiday in the context of preventing MRONJ.
